# Quantitative Software Analysis of Endoscopic Ultrasound Images of Pancreatic Cystic Lesions

**DOI:** 10.3390/diagnostics12092105

**Published:** 2022-08-30

**Authors:** Bánk Keczer, Márton Benke, Tamás Marjai, Miklós Horváth, Pál Miheller, Ákos Szücs, László Harsányi, Attila Szijártó, István Hritz

**Affiliations:** Department of Surgery, Transplantation and Gastroenterology, Semmelweis University, 1082 Budapest, Hungary

**Keywords:** endosonography, endoscopic ultrasonography, software analyzing, pancreatic cystic neoplasms, pseudocysts, pancreatic cysts

## Abstract

Endoscopic ultrasonography (EUS) is the most accurate imaging modality for the evaluation of different types of pancreatic cystic lesions. Our aim was to analyze EUS images of pancreatic cystic lesions using an image processing software. We specified the echogenicity of the lesions by measuring the gray value of pixels inside the selected areas. The images were divided into groups (serous cystic neoplasm /SCN/, intraductal papillary mucinous neoplasms and mucinous cystic neoplasms /Non-SCN/ and Pseudocyst) according to the pathology results of the lesions. Overall, 170 images were processed by the software: 81 in Non-SCN, 30 in SCN and 59 in Pseudocyst group. The mean gray value of the entire lesion in the Non-SCN group was significantly higher than in the SCN group (27.8 vs. 18.8; *p* < 0.0005). The area ratio in the SCN, Non-SCN and Pseudocyst groups was 57%, 39% and 61%, respectively; significantly lower in the Non-SCN group than in the SCN or Pseudocyst groups (*p* < 0.0005 and *p* < 0.0005, respectively). The lesion density was also significantly higher in the Non-SCN group compared to the SCN or Pseudocyst groups (4186.6/mm^2^ vs. 2833.8/mm^2^ vs. 2981.6/mm^2^; *p* < 0.0005 and *p* < 0.0005, respectively). The EUS image analysis process may have the potential to be a diagnostic tool for the evaluation and differentiation of pancreatic cystic lesions.

## 1. Introduction

Pancreatic cystic neoplasms (PCNs) affect a large percent of the general population. According to epidemiologic data they can be present in 2–45% of the population [1]. Because their biological behavior ranges from benign to malignant, the key to the accurate management is to prevent the advancement to pancreatic cancer, although to distinguish between the different types of pancreatic cystic neoplasms is often challenging [1,2,3].

Clinically, the most important PCNs are the serous cystic neoplasms (SCNs), intraductal papillary mucinous neoplasms (IPMNs), mucinous cystic neoplasm (MCNs) and solid pseudopapillary neoplasms (SPNs). The SCNs tend to be benign lesions with almost zero malignant potential while the IPMNs, MCNs and SPNs have a higher risk of malignant progression. Accordingly, the first choice of treatment is surgical resection before the cystic neoplasms evolve to a high-grade cancer. Due to the different malignant potential of PCNs, the key to the appropriate treatment is the thorough distinction between the SCN and Non-SCN lesions. The imaging modalities play a prime role in differentiation including the cross-section imaging methods, such as computed tomography (CT) and magnetic resonance imaging (MRI). Furthermore, the ultrasonography modalities, such as the transabdominal ultrasonography (US), which is often the first-choice imaging modality, and endoscopic ultrasonography (EUS), are critical elements of the diagnostic process and can provide crucial information increasing the diagnostic efficiency. Nevertheless, the precise and correct classification of the different types of PCNs based on their radio-morphological features remains a great challenge even for the most qualified radiologists. Due to the rapid development of Information Technology, the computer-aided diagnosis support is being used increasingly and may contribute to the decision-making process with a great significance. Several pieces of software were developed to enhance the diagnostic accuracy in different types of pathological abnormalities in various organs, such as the breast, lung and brain. However, in cases of pancreatic disorders only a few studies were published concerning the software-aided diagnostic decision [2,4,5].

Although, in terms of sensitivity, the EUS is superior compared to CT or MRI in the morphological assessment of PCNs, it is a semi-invasive and operator-dependent imaging modality. Furthermore, the EUS-guided fine-needle aspiration (FNA) or fine-needle biopsy (FNB) grants a greater possibility of distinguishing between the PCNs [6,7].

The increased incidence of PCNs could be explained by the greater awareness of their presence and particularly the wide use of cross-sectional imaging methods which results in the incidental detection of pancreatic cystic lesions. Earlier, the pseudocysts were held accountable for most of the pancreatic cysts but with the increased use of abdominal imaging in patients without a history of pancreatitis, it is becoming clear that PCNs are far more frequent than we previously thought [8].

Using CT or MRI alone in the diagnostic decision process may be useful when the typical radio-morphological features of the PCNs are present, but typically they appear only in the minority of the cases. It has been shown that CT or MRI were unsatisfying in the precise identification of the exact type of PCNs when the pre-operative radiological diagnosis was compared with the postoperative pathology [8]. The studies have also revealed that the cross-sectional imaging methods struggle to distinguish between the mucinous and the non-mucinous pancreatic cystic lesions [8]. Even though the EUS is a semi-invasive and operator-dependent imaging modality, it is able to visualize the PCN’s worrisome features (e.g., solid mass, mural nodules, main pancreatic duct dilatation and ductal communication and the exact size of the lesion) rich in details and with a higher resolution [2,8].

The aim of our study was to analyze the EUS images of pancreatic cystic lesions using an image processing software and furthermore, to find objective and quantitative attributions by the software assessment to distinguish between the PCNs with malignant potential and benign lesions.

## 2. Materials and Methods

We conducted a single center study from January 2018 to June 2021. The EUS (Olympus EU-ME2 GF-UCT180) with the right indication was performed in adult patients (age >18 years) with pancreatic cystic lesions (including PCNs and pseudocysts) previously detected by CT, MRI or transabdominal ultrasonography.

The patients were included into the study that was approved by the national Ethical Committee.

Fine needle aspiration (FNA) or biopsy (FNB) was performed if it was necessary for further differentiation. Three groups were created, based on the cytology (EUS-guided FNA or FNB with certain cytology results, questionable results were excluded) and/or the postoperative pathology results. The intraductal papillary mucinous neoplasms (IPMNs) and mucinous cystic neoplasms (MCNs) were classified as Non-SCN category as they both present with a higher risk of malignancy. The SCN group contained only SCN lesions, and the third group was comprised of the pseudocysts.

The images of the lesions were taken and saved for further analysis during the EUS examination. The procedures were performed by the same expert endosonographist. All of the PCNs were assessed with the same ultrasonography frequency (5 MHz) and the focus distance was also set to the same way. The pictures with the most worrisome features were taken and saved for further evaluation. More of the images were taken if the lesion could be also visualized from another position or angle with a different appearance. All of the pictures were saved in the same resolution (1280 × 960) and in the same format (jpg.). All of the images were calibrated (12.2674 pixels/mm) based on the ultrasonography distance scale and analyzed in 8 bit which meant 256 possible gray tonal values for each pixel.

The mean gray value of the lesions meant the mean of the selected areas pixels’ gray value in an 8 bit image format which corresponded to the echogenicity of the lesions. The standard deviation of the lesions meant the standard deviation of the selected areas pixels’ gray value in 8 bit image format which corresponded to the inhomogeneity of the lesions. The density was a standardized characteristics’ value for the lesions. It meant the sum of the gray values of the pixels in the selected part, divided by the selected part’s area value:

$$Density=\frac{\sum Gray\;values\;in\;the\;selected\;part}{Selected\;part^{’}s\;Area}$$

The area values in mm^2^ were calculated based on the calibrated estimates. The image requirements were as follows: image saved in the correct format, EUS performed on 5 MHz, lesion well defined, all parts of the lesions assessable and no duplication image of the same lesion.

**Figure 1:**
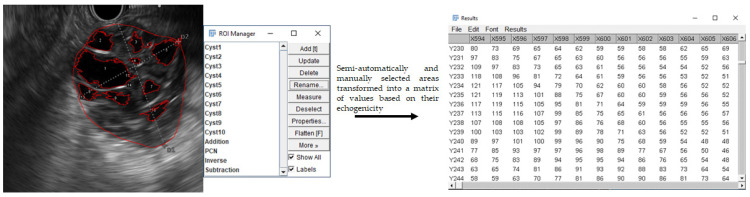
Image analysis method.

We specified the echogenicity of the lesions by measuring the gray value of the pixels inside the selected areas. Besides the entire lesion, its cystic and solid parts (e.g., intracystic septa, nodules, cystic wall) were also selected separately for assessment. The entire lesion area was selected manually, using the software’s free hand selection feature. The cystic parts were selected semi-automatically with the software’s tracing tool where a tolerance value could be set in advance. Selecting a pixel inside the cystic region after the correct tolerance settings, the software automatically also selected the surrounding pixels based on the preceding tolerance setting. The tolerance setting determined the permissible gray value difference between the selected pixel and the automatically selected surrounding ones. The values of the solid parts were calculated by a mathematical formula, during which the values of the cystic part(s) were subtracted from the values of the whole lesion. Thus, the solid parts (e.g., the cystic wall of the Pseudocysts or SCNs) could be determined and measured much more accurately than with the free hand selection feature (Figure 1).

To standardize the analysis process, beside the cystic lesions, a predetermined area (three circles with a diameter of 5 mm each) of healthy pancreatic parenchyma was also selected for assessment. All of these selected areas’ echogenicity showed no significant differences compared to each other’s, therefore every echogenicity value of the healthy pancreas originated from the same sample population.

The exclusion criteria were as follows: unavailable postoperative pathology or unclear FNA/FNB cytology result; images saved in the wrong format; lesion not well-defined or not all parts of the lesion assessable.

GraphPad Prism, SPSS and Microsoft Excel software were used for the data evaluation. Normality tests (Anderson–Darling, D’Agostino and Pearson, Shapiro–Wilk, Kolmogorov–Smirnov) were performed to determine the samples’ normality level. In cases of normal distribution, the independent samples *t*-test, otherwise the non-parametric independent test, was applied. In some cases, the Χ^2^ test was performed. A *p* value < 0.05 was considered statistically significant. The FIJI software was used for image analyzing [9].

## 3. Results

**Figure 2:**
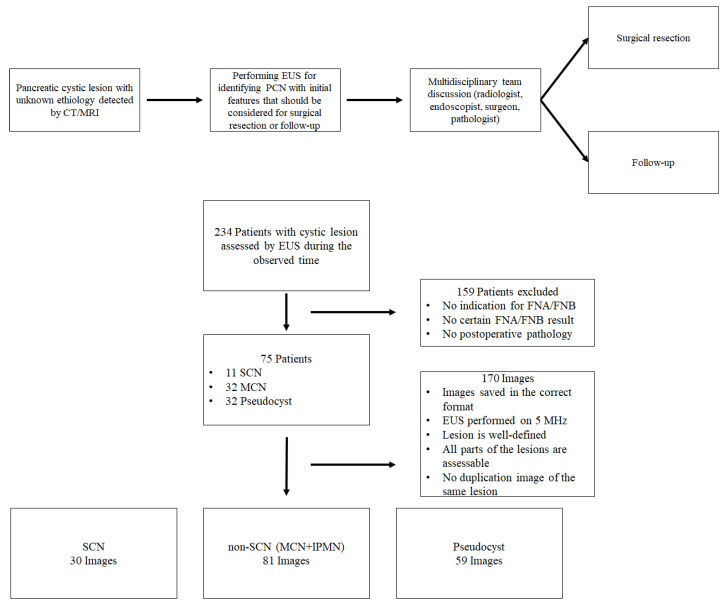
Inclusion and exclusion criteria of patients and evaluation method. Diagnostic flowchart.

During the observed period, 234 patients with suspected PCN or pseudocysts underwent EUS examination. The correct cytology reports from FNA or FNB sampling and the postoperative pathology results were available in 75 patients. A total of 170 images were processed by the image analyzing software. In the SCN group, 30 images (11 patients) met the requirements for the software analysis (described in the Methods section), while in the Non-SCN group 81 images (32 patients) and in the Pseudocyst group 59 images (32 patients) could be assessed, respectively (Figure 2).

The mean age of the patients in the SCN, Non-SCN and the Pseudocyst groups were 59.4 ± 20.1, 61.8 ± 11.5 and 63.3 ± 13.1 years, respectively. In the SCN group 82% (9/11) of the patients were female, while in the Non-SCN and Pseudocyst groups 72% (23/32) and 63% (20/32), respectively. There were no significant differences between the groups in terms of age and gender (Table 1).

The mean echogenicity of the healthy pancreas parenchyma was 68.9 ± 10.4, 68.3 ± 11.3 and 69.4 ± 11.1, respectively, in the SCN, Non-SCN and Pseudocyst groups. There was no significant difference between the groups.

**Figure 3:**
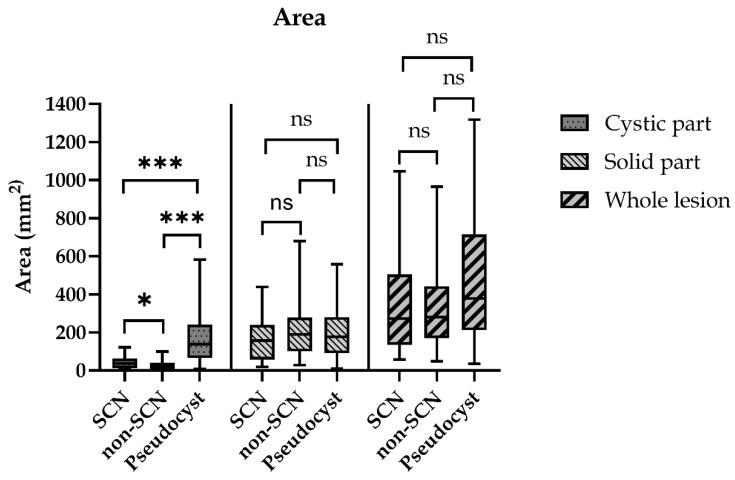
Areas of the different parts of the cystic lesions. Area values were calculated by the software based on the selected areas. (ns = non-significant; * *p* < 0.05; *** *p* < 0.0005).

The whole lesions’ mean sizes were 415.8 ± 64.2 mm^2^, 433.2 ± 47.4 mm^2^ and 590.4 ± 77.6 mm^2^, respectively, in the SCN, Non-SCN and the Pseudocyst groups. There was no significant difference between the SCN, Non-SCN and the Pseudocyst groups in terms of the lesion’s size. The cystic parts’ mean size were 116.8 ± 25.4 mm^2^, 74.9 ± 14.1 mm^2^ and 324.1 ± 55.8 mm^2^, respectively, in the SCN, Non-SCN and the Pseudocyst groups. In the Non-SCN group, the cystic parts’ areas were significantly smaller than in the SCN or the Pseudocyst groups (*p* = 0.013 and *p* < 0.0005, respectively). The Pseudocysts’ cystic area was significantly larger than the SCNs’ size (*p* < 0.0005) (Figure 3).

The mean number of cystic lobules of the lesions was 2.1 in the SCN group, 2.4 in the Non-SCN and 1.2 in the Pseudocyst groups. There was no significant difference between the SCN and the Non-SCN groups, but in the Pseudocyst group the lesions had significantly fewer cystic parts, compared to both of the other groups (*p* < 0.0005) The mean value of the area ratio, which meant the proportion of the cystic part to the whole lesion was 57%, 39% and 61%, respectively, in the SCN, Non-SCN and the Pseudocyst groups. There was no significant difference between the SCN and Pseudocysts groups, but the Non-SCN group’s area ratio was significantly lower than the SCN and Pseudocyst groups (*p* < 0.0005).

**Figure 4:**
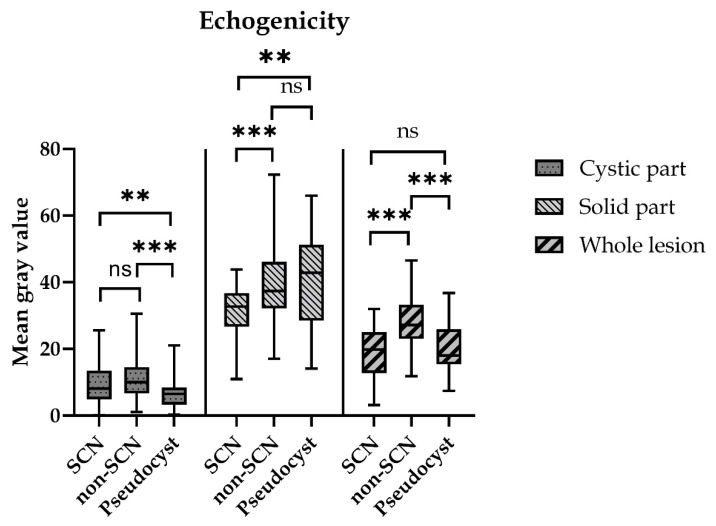
Mean gray values of the different parts of cystic lesions. Based on the echogenicity, the lesions were transformed into a matrix of values and the average gray value of the areas was determined by the software. (ns = non-significant; ** *p* < 0.005; *** *p* < 0.0005).

The mean gray value of the whole lesions was 18.8 ± 1.2, 27.8 ± 0.9 and 19.8 ± 0.9, respectively, in the SCN, Non-SCN and Pseudocyst groups. The mean gray value in the Non-SCN group was significantly higher (*p* < 0.0005) compared to both of the other groups. There was no significant difference between the SCN and the Pseudocyst groups. The mean gray value of the cystic parts was 9.7 ± 0.7, 11.1 ± 0.4 and 7.5 ± 0.7, respectively, in the SCN, Non-SCN and Pseudocyst groups. In the Pseudocyst group, the mean gray value of the cystic parts was significantly lower than in the Non-SCN (*p* < 0.0005) and in the SCN (*p* = 0.007) groups. The mean gray value of the solid parts (intracystic septa, mural nodules, cystic walls) was higher in the Non-SCN (39.0 ± 1.2) and in the Pseudocyst (40.7 ± 1.6) groups than in the SCN group (31.4 ± 1.2). There was no significant difference between the Non-SCN and Pseudocyst groups, but in the SCN group, the cystic mean gray value was significantly lower compared to the Non-SCN (*p* = 0.0009) and the Pseudocyst (*p* < 0.0017) groups (Figure 3 and Figure 4).

**Figure 5:**
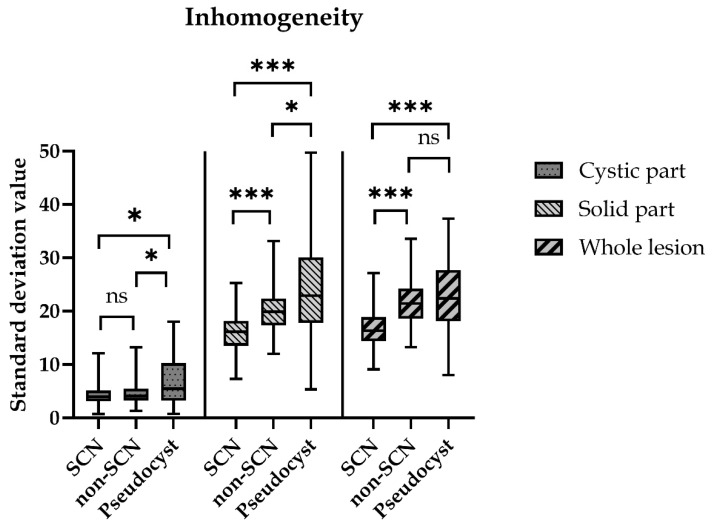
Inhomogeneity values of the different parts of cystic lesions. The inhomogeneity of the lesions was measured by the difference in the gray value of the pixels of the selected areas, which was the standard deviation of the gray value of the selected area. (ns = non-significant; * *p* < 0.05 *** *p* < 0.0005).

The inhomogeneity value of the whole lesion was 16.6 ± 0.7 in the SCN, 22.3 ± 0.6 in the Non-SCN and 22.6 ± 0.8 in the Pseudocyst group. The inhomogeneity value was significantly higher in the Non-SCN (*p* < 0.0005) and in the Pseudocyst (*p* < 0.0005) groups than in the SCN group. There was no significant difference between the Non-SCN and the Pseudocyst groups. There was no significant difference between the inhomogeneity of the cystic parts, the values were 7.1 ± 0.7, 6.9 ± 0.3 and 7.1 ± 0.6, respectively, in the SCN, Non-SCN and Pseudocyst groups. However, the solid parts’ inhomogeneity was significantly higher in the Non-SCN (21.1 ±0.6; *p* < 0.0005) and in the Pseudocyst (24.3 ± 1.1; *p* < 0.0005) groups than in the SCN (16.1 ± 0.7) group. The calculated value was significantly higher in the Pseudocyst group compared with the Non-SCN group (*p* = 0.017) (Figure 5).

**Figure 6:**
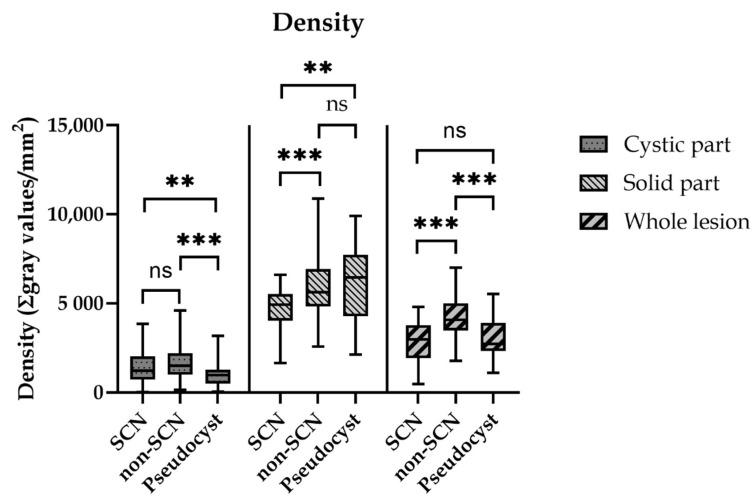
Density values of the different parts of cystic lesions. The density value meant the quotient of the sum of gray values in the selected area and the value of the selected area (ns = non-significant ** *p* < 0.005; *** *p* < 0.0005).

The density of the whole lesions in the SCN, Non-SCN and Pseudocyst groups was 2833.8/mm^2^ ± 192.6, 4186.6/mm^2^ ± 135.6 and 2981.6/mm^2^ ± 144.5, respectively. The density of the Non-SCN group was significantly higher than in the SCN (*p* < 0.0005) and the Pseudocyst (*p* < 0.0005) groups. There was no significant difference between the SCN and the Pseudocyst groups. The density of the cystic parts in the SCN, Non-SCN and Pseudocyst groups was 1461.4/mm^2^ ± 117.0, 1668.6/mm^2^ ± 67.57 and 1127.3/mm^2^ ± 107.1, respectively. In the Pseudocyst group, the density was significantly lower than in the SCN (*p* = 0.015) and in the Non-SCN (*p* < 0.0005) groups. There was no significant difference between the SCN and the Non-SCN groups. The solid parts’ density was highest in the Pseudocyst group (6117.9/mm^2^ ± 25.6), while it was lowest in the SCN group (4737.6/mm^2^ ± 194.6), meanwhile, in the Non-SCN group it was 5875.0/mm^2^ ± 183.0. The density of the SCN’s solid part was significantly lower than in the Non-SCN (*p* = 0.0009) and the Pseudocyst (*p* < 0.005) groups. There was no significant difference between the Non-SCN and the Pseudocyst groups (Figure 6).

## 4. Discussion

EUS as an imaging modality has many advantages, including the detailed visualization of the walls of internal organs corresponding to histological layers, high diagnostic yield in analyzation of adjacent structures, the possibility of real-time guided FNA/FNB and has the greatest sensitivity in detecting small lesions in the pancreas, though at the same time it is a highly operator- and skill-dependent, semi-invasive method with a long-lasting learning curve to master.

The results, and thus the patient’s clinical outcome, often depend on the practitioner’s skill level. The differentiation of cystic lesions based on their EUS morphology alone is limited, accordingly the benign, malignant or even inflammatory lesions may have indistinguishable appearance. EUS-guided sampling (EUS-FNA or FNB) can improve the diagnostic accuracy. Recent studies showed that EUS-FNA has 91% sensitivity and 94% specificity for solid pancreatic neoplasms but performs worse in the diagnosis of cystic pancreatic neoplasms with a sensitivity and specificity of 54% and 93%, respectively, and with a high positive likelihood ratio for MCNs. The combination of EUS morphology with EUS-guided tissue sampling analysis and the intracystic carcino-embryogenic antigen (CEA) levels guarantees the best diagnostic accuracy [6,10,11,12,13,14,15].

The groundbreaking developments in the field of Information Technology in recent years provide great possibilities for assisting in the decision-making process based on real quantitative data in substitution for the skill-dependent and subjective estimates and providing more accurate results [13]. The spread of software image analysis in medicine is creating new opportunities that can improve the diagnostic efficacy in all areas of the profession [16]. The analysis of EUS images may facilitate the diagnosis of cystic lesions of the pancreas and may also facilitate the treatment decision by characterizing the lesions’ morphology based on quantitative data.

In our study there was not a significant difference between the groups in terms of gender ratio although the majority of patients in both the SCN and Non-SCN groups were female while the Pseudocyst group was less female dominant. These epidemiologic data correlate with those observed earlier [17]. The age distribution did not show any significant difference.

The assessed size of the whole lesions by EUS did not show significant differences, although the Pseudocysts were slightly bigger. The etiology of our observed lesions was unspecified due to the fact that most of them were incidentally discovered by different imaging methods. The follow-up fluid lesions after pancreatitis, such as Pseudocysts, can achieve an enormous size, nevertheless these lesions were excluded from our study because of their known etiology. The SCN lesions were of an oligocystic or unilocular type. The microcystic-type SCNs lesion might be identified as solid lesions, as their cystic parts sometimes are too small even on the EUS. The majority of the indications of pancreatic resections is based predominantly on the lesions’ size, however studies showed that a high proportion of invasive MCNs are smaller than 4 cm which draws attention to a stricter follow-up approach with objectively measurable morphology features besides the lesions’ size [18].

We found that the Pseudocyst lesions had usually one major cystic lobule while the SCN and Non-SCN lesions usually had more than two. There was no significant difference between the number of cystic lobules between the SCN and Non-SCN groups, in contrast with the Pseudocyst group. It has been assumed that the microcystic SCN lesions with a typical morphology might have more cystic lobules, in addition these lesions are usually identified by MRI or CT, so there is no need for further EUS assessment. However, about 30% of the SCN lesions show a non-classical appearance, thus the oligocystic, unilocular or microcystic SCNs with atypical morphology can be deceptive even for an experienced eye, which can lead to a remarkable number of unnecessary surgeries. Pancreatic resection is not indicated in all cases of pancreatic cystic lesions since it can be associated with serious complications and perioperative morbidity and mortality [19]. Keeping in mind the principles of the step-up approach and cost–benefit risk assessment, with the majority of cystic lesions it is just better to follow up. While differentiating the typical SCN from MCN in the pre-operative diagnostics is relatively unchallenging, the differential diagnosis between the atypical SCN and MCN is much more challenging [19].

The proportion of cystic part(s) to the whole lesion was largest in the Pseudocyst group. The Non-SCN group typically presented with more cystic parts, but the cysts’ size was less, thus the area ratio was the lowest among the groups.

The lesions’ echogenicity was calculated through the gray mean values. The whole lesion echogenicity was most significant in the Non-SCN group which correlated with the area ratio. The greater the proportion of solid parts the lesion had, the more of the gray mean values of the whole lesion were calculated. The value of the solid parts was highest in the Pseudocyst group because pseudocysts usually present with thick and hyperechogenic cystic walls, which can be explained by their fibrotic cyst wall histology. The solid parts of the Non-SCN lesions were less echogenic, essentially due to the MCN lesions’ mucin-producing epithelium supported by ovarian-type stroma, while the SCN lesions’ value was the lowest because they are characterized only by the epithelium [19].

In summary, the whole lesion’s sizes did not show significant differences, but the sizes of the cystic parts did. The current guidelines consider only the whole lesion’s size and do not make a difference between the lesion’s area ratio (pseudocysts mostly have only cyst wall while MCNs have more solid parts). Other worrisome features such as thickened or enhancing cyst walls are also not quantified and standardized in the guidelines, making these features difficult to interpret. The measurement of echogenicity of the different parts of the lesions in our study showed that these parameters are easily quantifiable; the notable differences might be used in the clinical decision-making process [1].

Augmenting the diagnostic yield of EUS with objective and easily quantifiable values which are not visible to the naked eye can improve its diagnostic accuracy and enhance the sensitivity. Moreover, improving the pre-operative diagnostic accuracy can prevent further unnecessary procedures and interventions resulting in a more precise and cost-efficient decision-making process, thus optimizing the clinical outcome [10,11].

The EUS image processing analysis may have the potential to be a diagnostic novel tool for the evaluation and differentiation of pancreatic cystic lesions, furthermore, it might have the potential to broaden the role of artificial intelligence in the diagnosis of gastrointestinal disorders, albeit more comprehensive research is recommended to verify the clinical efficacy and accuracy of the EUS image analyzing method. Our study has its own limitations. Endoscopic ultrasonography is a subjective, operator-dependent diagnostic method; however, in expert hands, it represents and guarantees the most sensitive diagnostic tool, which is capable of showing the most detailed images of the lesions. The number of images was limited by the incidence of cystic lesions in patients who underwent EUS.

## Figures and Tables

**Table 1:** Groups and values.

Groups		Pseudocyst	SCN	Non-SCN
Number of images (*n*=)		59	30	81
CT/MRI performed before EUS		100%	100%	100%
Biopsy/postoperative diagnosis		75%/25%	64%/36%	44%/56%
Multidisciplinary team decision		100%	100%	100%
Echogenicity of healthy pancreatic parenchyma		69.4 ±11.1 SD	68.9 ± 10.4 SD	68.3 ± 11.3 SD
Number of cystic lobules (*n*=)		1.2	2.1	2.4
Area ratio		61%	57%	39%
Area (mm^2^)	Cystic part	324.1 ± 55.8 mm^2^	116.8 ± 25.4 mm^2^	74.9 ± 14.1 mm^2^
	Solid part	196.1 ± 131.6 mm^2^	168.2 ± 122.5 mm^2^	248.5 ± 199.9 mm^2^
	Whole lesion	590.4 ± 77.6 mm^2^	415.8 ± 64.2 mm^2^	433.2 ± 47.4 mm^2^
Echogenicity	Cystic part	7.5 ± 0.7	9.7 ± 0.7	11.1 ± 0.4
	Solid part	40.7 ± 1.6	31.4 ± 1.2	39.0 ± 1.2
	Whole lesion	19.8 ± 0.9	18.8 ± 1.2	27.8 ± 0.9
Inhomogeneity	Cystic part	7.1 ± 0.6	7.1 ± 0.7	6.9 ± 0.3
	Solid part	24.3 ± 1.1	16.1 ± 0.7	21.1 ±0.6
	Whole lesion	22.6 ± 0.8	16.6 ± 0.7	22.3 ± 0.6
Density (∑gray values/mm^2^)	Cystic part	1127.3/mm^2^ ± 107.1	1461.4/mm^2^ ± 117.0	1668.6/mm^2^ ± 67.5
	Solid part	6117.9/mm^2^ ± 25.6	4737.6/mm^2^ ± 194.6	5875.0/mm^2^ ± 183.0
	Whole lesion	2981.6/mm^2^ ± 144.5	2833.8/mm^2^ ± 192.6	4186.6/mm^2^ ± 135.6

## Data Availability

Not applicable.
